# Re-evaluating Renal Angina Index: An Authentic, Evidence-Based Instrument for Acute Kidney Injury Assessment: Critical Appraisal

**DOI:** 10.3389/fped.2021.682672

**Published:** 2021-07-29

**Authors:** Rupesh Raina, Sidharth Kumar Sethi, Isabelle Mawby, Nikhil Datla, Nikhita Kumar, Nirav Agarwal, Abhishek Tibrewal, Ronith Chakraborty

**Affiliations:** ^1^Department of Nephrology, Akron Nephrology Associates/Cleveland Clinic Akron General Medical Center, Akron, OH, United States; ^2^Department of Nephrology, Akron Children's Hospital, Akron, OH, United States; ^3^Pediatric Nephrology, Kidney Institute, Medanta, The Medicity Hospital, Gurgaon, India; ^4^Department of Medicine, Northeast Ohio Medical School, Rootstown, OH, United States; ^5^Feinstein Institutes for Medical Research, Northwell Health, Manhasset, NY, United States

**Keywords:** renal angina index, pediatric, acute kidney injury, intensive care unit, indicator

## Abstract

**Background/Introduction:** Renal angina index (RAI) used to calculate and accurately predict risk for the development of acute kidney injury (AKI) has been heavily explored. AKI is traditionally diagnosed by an increase in serum creatinine (SCr) concentration or oliguria, both of which are neither specific nor sensitive, especially among children. An RAI score may be calculated by combining objective signs of kidney dysfunction (such as SCr) and patient context, such as AKI risk factors, thus potentially serving as a more accurate indicator for AKI.

**Objective:** Due to the propitious and novel nature of RAI, this editorial commentary aims to analyze the current literature on RAI and determine how well RAI serves as a predictor of AKI outcomes.

**Method:** A comprehensive literature search was conducted in PubMed/Medline and Google Scholar between January 2012 and July 2020. Literature included the prognostic aspect of early prediction of AKI in the pediatric and adult population via RAI.

**Results:** The initial literature search included 149 studies, and a total of 10 studies reporting the outcomes of interest were included. The overall sample size across these studies was 11,026. The predictive ability of RAI had a pooled (95% CI) sensitivity of 79.21%, specificity of 73.22%, and negative predictive value of 94.83%.

**Conclusion:** RAI shows benefit in the prediction of AKI among adult and pediatric populations. However, there is a lack of sufficient data, and further prospective studies are needed in pediatric populations to use RAI as a principal AKI indicator among clinicians.

## Introduction

Acute kidney injury (AKI) is a common condition associated with high morbidity and mortality rates in critically ill patients. It is characterized by a consortium of conditions including a sudden decrease in glomerular filtration rate (GFR), thus a decline in the kidney's excretory function. Clinically, AKI is diagnosed using the Kidney Disease: Improving Global Outcomes (KDIGO) guidelines by an increase in serum creatinine (SCr) or oliguria (1, 2). The severity of AKI is classified into three stages based on SCr level and urine output and is independently associated with increased patient morbidity and mortality (3). In any case, SCr levels have a high variability among children and cannot accurately predict AKI (4), which is why renal angina index (RAI) was originally proposed (3). Thus, an improvement upon SCr through the use of RAI will serve to better detect and predict kidney injury, especially among pediatric populations where SCr has shown to be even less precise (4). Additionally, there is no singular and effective therapy for AKI that exists, and management consists of supportive care; therefore, it is imperative to explore all the options for early diagnosis and preventive measure of this common clinical problem (1).

In 2012, Basu et al. (5) proposed a scoring system, RAI, as a predictor of AKI in critically ill children. This scoring system was formulated based on two variables, AKI risk levels (high-risk procedures such as bone marrow transplant, which was scored based on the risk level; five points for patient on ventilation or cardiac surgery, three points for nephrotoxic drugs or burn injuries, and one point for trauma or sepsis) and evidence of AKI injury (increase SCr, oliguria, and fluid overload), which was similarly scored based on % FO. Both AKI risk levels and AKI injury levels are multiplied to get the RAI score (Table 1). Higher predictability of AKI was seen when AKI risk factors and evidence were multiplied, resulting in an increased score (≥8). The index was designed to have an extremely high negative predictive value (NPV) to eliminate all the patients who are not likely to develop AKI (5).

**Table 1:** Risk calculation.

**Risk level**	**Risk**	**Score**
Trauma/sepsis	Moderate	1
Nephrotoxic drugs/burn injuries	High	3
Ventilation/cardiac surgery	Very high	5
**Injury level**	**%FO**	**Score**
No change	<5	1
0−24%	≥5	2
25−49%	≥10	4
≥50%	≥15	8

Renal angina index is calculated using the following formula: Renal Angina = risk of AKI ^*^ signs of injury (1–40). Acute kidney injury (AKI) incidence increases as the score increases. Adapted from Basu et al. (6).

In 2014, Basu et al. (6, 7) conducted a multicenter cohort study to discuss the use of RAI and its significance in the early diagnosis of AKI. A total of 584 patients were analyzed with a sensitivity of 58–93% and NPV of 92–99% across all the included study sites. RAI had a higher sensitivity and specificity than neutrophil gelatinase-associated lipocalin (NGAL), matrix metalloproteinase-8 (MMP-8), and neutrophil elastase-2 Ela-2, all serum biomarkers for prediction of severe AKI (>200% increase in SCr) (6). Furthermore, RAI was compared with KDIGO staging and Pediatric Risk of Mortality (PRISM II), and it demonstrated a significantly higher pretest probability (6).

Irrespective of the above study's promising results, there were few limitations including a smaller sample size and utilization of the standard SCr values (based on patients' height and age) in the absence of baseline SCr values. Therefore, this systematic review aims to assess all the available literature to evaluate the use of RAI as an indicator for early detection of AKI in the pediatric population.

## Method

A literature search was conducted in PubMed/Medline and Google Scholar. The search consists of the following medical subject heading (MeSH) terms, “Renal Angina Index,” “Acute kidney injury,” “RAI,” “Intensive Care units,” “Acute Renal Failure,” “Neonatal Intensive Care Unit,” “Urine output,” “Fluid overload,” “Serum creatinine,” and “Glomerular filtration rate,” in various combinations. All the articles were limited to the English language and newborn to 18 years old. The literature search was conducted between January 2012 and July 2020.

Studies were selected by two independent reviewers. When there are any conflicts during the selection process, the third investigator's opinion was considered. A PICO (Patient Population, Intervention, Control, Outcome) table was used to describe the study criteria (Supplementary Table 1). After the selection process, a summary table was created, extracting individual information from the included studies: the last name of the first author, study type, total population group, patient demographic (age, male, and female), patient characteristics, total number of patients with AKI with RAI score of >8, total number of patients with AKI, and study outcome (Table 1). A Preferred Reporting Items for Systematic Reviews and Meta-Analyses (PRISMA) flowchart was created mentioning the inclusion and exclusion studies (Supplementary Figure 1). This systematic review was performed according to the PRISMA Checklist (Supplementary Table 2).

### Quality Assessment

A quality assessment table was created to verify all the included studies in the systematic review. We used National Heart, Lung, and Blood Institute (NHLBI) (https://www.nhlbi.nih.gov/health-topics/study-quality-assessment-tools) assessment tool, “Quality assessment tool for Observational Cohort and Cross-Sectional Studies” to assess the included studies. There were a series of 14 questions, and the reviewer responded to each question with “yes,” “no,” and “not applicable/not reported/cannot determine.” Furthermore, each “yes” received one point, and the total score was added under the “Overall Outcome” column. Studies with a score between 12 and 14 are considered good quality, scores between 9 and 11 are fair quality, and a score below nine is considered a poor-quality study (Supplementary Table 3).

### Statistical Analysis

The outcomes included pretest predictability of RAI [sensitivity, specificity, positive predictive value (PPV), NPV, and area under the curve (AUC)] and mortality among RAI >8 vs. RAI <8 patients (secondary outcome). Summary receiver operating characteristic (SROC) curves were plotted with the presentation of a summary operating point, 95% confidence, and prediction contours. These outcomes and its 95% confidence intervals (95% CIs) were computed (calculated when not reported) for each study. Diagnostic odds ratio (95% CI) was calculated using the following formula: (sensitivity × specificity)/[(1 – sensitivity) × (1 – specificity)]. Positive and negative likelihood ratios (LRs) (95% CI) were calculated using the formulas sensitivity/(1 – specificity) and (1 – sensitivity)/specificity, respectively. The degree of between-study heterogeneity was assessed using the I^2^ test, where *I*^2^ ≥ 50% indicated high heterogeneity. Overall (pooled) estimate was calculated with random effects model for high heterogeneity and fixed effects model for low heterogeneity. To determine the source of heterogeneity, sensitivity analyses were performed based on these parameters (study design, sample size of the study, and study quality). Forest plot was used to visualize these outcomes in each study and the combined estimated outcomes with their 95% CI. Publication bias was assessed with a funnel plot and Egger's test. Egger's linear regression test was used to evaluate asymmetry. A *p*-value ≤ 0.05 was set as the level of significance. All statistical analyses were performed with R software version 3.1.0.

## Results

### Included Studies

A total of 10 studies were analyzed. Of these, seven were prospective and three were retrospective studies (Table 2). The overall sample size across these studies was 11,026 (RAI > 8 = 2,513 and RAI < 8 = 8,513). The median age of the patients across these studies was 4.5 years (2.0–10.5 years), and 57.4% (n = 6,325) were male. Our quality assessment tool identified seven studies as good quality and three studies as fair quality. Types of studies included prospective observational cohort, retrospective observational, and multicenter retrospective cohort studies. These studies had a minimum number of 53 patients and a max of 7,505 giving this meta-analysis a good variety of applicable information.

**Table 2:** Summary of all the included studies for RAI in pediatric population.

**References**	**Type of study**	**No. of patients (N)**	**Age (years)**	**Outcome**
Gawadia et al. (8)	Prospective observational cohort	162	10.5	RAI when ≥8 on the first day of hospitalization, reliably identifies those critically ill children who are at higher risk for developing severe AKI on day 3 of hospitalization
Hanson et al. (9)	Prospective observational cohort	118	7.8 ± 6.4	In this pilot study, RAI was shown to be a sensitive test that can be used in the ED and that outperforms using a change in SCr to predict AKI 24 h after admission to the hospital. This test has promise for potential application into the electronic medical record as an automated trigger tool
Kaur et al. (10)	Prospective observational cohort	413	5.89 ± 5.31	RAI could be used as a simple and important bedside tool to predict patients at risk of severe AKI
Raman et al. (11)	Single-center retrospective observational study	7,505	2.0 (0.4–7.3)	The prevalence of AKI in our cohort is lower compared with that of other reports. Given the association between HRA and AKI, the development of a bedside tool that only needs a single baseline creatinine value and the age of the child to predict the probability of AKI at admission is exciting. If externally validated, especially in the non-cardiac population, clinicians may now have a tool for risk stratification
Zeid et al. (12)	Prospective observational	53	2.15	The study suggested that RAI has significant predictability for severe AKI, but incorporation of uNGAL into RAI improves detection ability of severe AKI
Basu et al. (3)	Observational study	1,590	4.5	Renal angina index predicted the risk of severe AKI better than SCr alone
Basu et al. (5)	Multicenter study	214	3.8 (1.6, 6.8) for RAI– and 1.7 (0.5, 5) years for RAI+	RAI optimizes the utility of AKI biomarkers in critically ill patient. Incorporation of AKI biomarkers into the RAI improves discrimination for severe AKI
Sethi et al. (13)	Prospective study	102	6.5 ± 5.9	The study focuses on the adverse effects of the positive fluid balance and emphasizes the use of RAI in clinical AKI identification
Sundararaju et al. (14)	Prospective observational study	285	4.4 (0.5–7.7)	The study concluded the usefulness of RAI in predicting the developments of severe AKI on days 3 and 7
Roy et al. (15)	Retrospective study	100	7.5 (2.1–14)	N/A
Basu et al. (6)	Multicenter retrospective cohort study	584	3.8 (1.2–12.5)	This cohort study was the first study to analyze the significance of use of RAI and its potentially to reduce the risk of future severe AKI

*AKI, acute kidney injury; SCr, serum creatinine; ED, emergency department; HRA, high risk of acute kidney injury; RAI, renal angina index; uNGAL, urine neutrophil gelatinase-associated lipocalin*.

### Predictive Ability of Renal Angina Index

**Figure 1:**
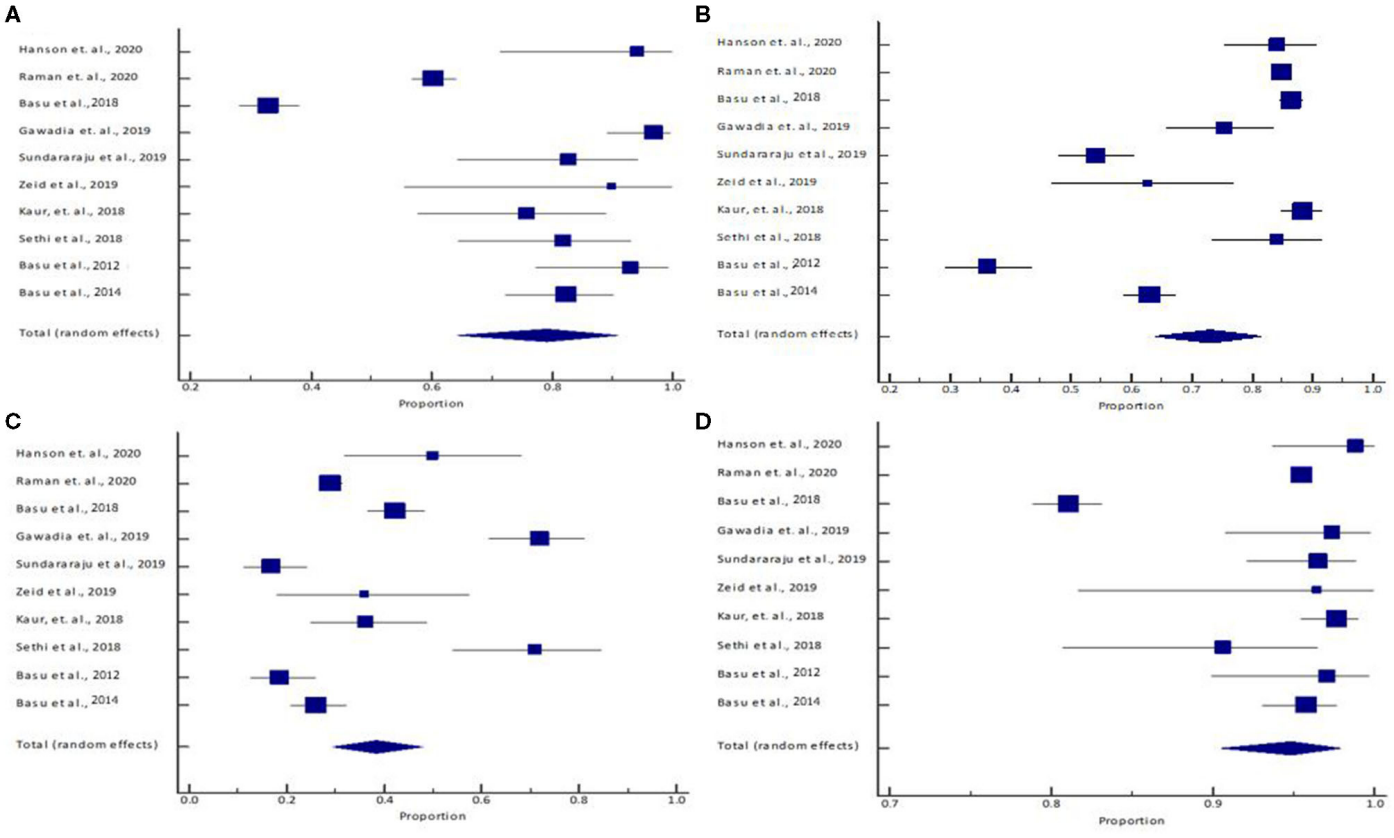
**(A)** Sensitivity, **(B)** specificity, **(C)** positive predictive value, and **(D)** negative predictive value. The lower diamond in the graphs represents the pooled estimate.

The pooled (95% CI) sensitivity: 79.21% (95% CI: 64.28–90.90%) [*I*^2^ = 96.33% (94.75–97.44%), *p* < 0.0001, random effects, 10 studies, n = 1,353] (Table 3; Figure 1A); specificity: 73.22% (95% CI: 64.13–81.42%) [*I*^2^ = 97.99% (97.30%−98.51%), *p* < 0.0001, random effects, 10 studies, *n* = 9,673] (Table 3; Figure 1B); PPV: 38.38% (95% CI: 29.37–47.81%) [*I*^2^ = 93.65% (90.28–95.85%), *p* < 0.0001, random effects, 10 studies, *n* = 2,513] (Table 3; Figure 1C); and NPV: 94.83% (95% CI: 90.49–97.91%) [*I*^2^ = 96.61% (95.19–97.62%), *p* < 0.0001, random effects, 10 studies, *n* = 8,513] (Table 3, Figure 1D) was observed for the RAI. The pooled (95% CI) AUC of RAI was found to be 0.85 (95% CI: 0.80–0.89) [*I*^2^ = 64.56% (14.60–85.29%); *p* < 0.0001; random effects; six studies; *n* = 1,776] (Table 3; Supplementary Figure 2). Supplementary Figure 3 shows the SROC graph of 10 included studies based on the random effects model. The diagnostic odds ratio (95% CI) based on pooled sensitivity and specificity values was calculated to be 10.45 (3.22–43.77). The DOR >1 suggests that the test is discriminating correctly. For our study, the DOR was 10.45, signifying a strong association between RAI and the predictability of early AKI. The positive and negative LRs (with 95% CI) were 2.96 (1.70–4.89) and 0.28 (0.11–0.56), respectively. The LR indicates how likely a patient has a disease or condition; and the higher the value, the more likely the patient has the condition. Positive LR describes how probability of disease shifts when the finding is present; negative LR describes how probability of disease shifts when it is absent (16). For our study, the positive LR was 2.96, indicating that RAI >8 test increases the pretest probability (prevalence; is based on the probability of the suspected disease in the person given their symptoms) of AKI by 20%, while the negative LR was 0.28, indicating that RAI <8 test decreases the pretest probability of AKI by 25%.

**Table 3:** Meta-analysis of sensitivity, specificity, positive predictive value, negative predictive value, and area under the curve.

**Study**	**True positive/disease positive**	**Sensitivity % (95% CI)**	**Random weight (%)**
**(a) Sensitivity**			
Hanson et al. (9)	16/17	94.12 (71.31–99.85)	9.14
Raman et al. (11)	417/690	60.44 (56.68–64.1)	11.02
Basu et al. (3)	121/368	32.88 (28.10–37.94)	10.97
Gawadia et al. (8)	62/64	96.88 (89.16–99.62)	10.47
Sundararaju et al. (14)	24/29	82.76 (64.23–94.15)	9.83
Zeid et al. (12)	9/10	90.00 (55.50–99.75)	8.23
Kaur et. al. (10)	25/33	75.76 (57.74–88.91)	9.96
Sethi et al. (13)	27/33	81.82 (64.54–93.02)	9.96
Basu et al. (5)	27/29	93.10 (77.23–99.15)	9.83
Basu et al. (6)	66/80	82.50 (72.39–90.09)	10.58
Total (random effects)	794/1,353	79.21 (64.28–90.90)	100
**Study**	**True negative/disease negative**	**Specificity % (95% CI)**	**Random weight (%)**
**(b) Specificity**			
Hanson et al. (9)	85/101	84.16 (75.55–90.67)	9.71
Raman et al. (11)	5,793/6,815	85.00 (84.13–85.84)	10.7
Basu et al. (3)	1,057/1,222	86.50 (84.45–88.37)	10.63
Gawadia et al. (8)	74/98	75.51 (65.79–83.64)	9.68
Sundararaju et al. (14)	139/256	54.30 (47.98–60.51)	10.29
Zeid et al. (12)	27/43	62.79 (46.73–77.03)	8.63
Kaur et. al. (10)	336/380	88.42 (84.77–91.46)	10.43
Sethi et al. (13)	58/69	84.06 (73.26–91.76)	9.3
Basu et al. (5)	67/185	36.22 (29.29–43.59)	10.14
Basu et al. (6)	318/504	63.10 (58.72–67.32)	10.5
Total (random effects)	7,954/9,673	73.22 (64.13–81.42)	100
**Study**	**True positive/test positive**	**PPV % (95% CI)**	**Random weight (%)**
**(c) Positive predictive value (PPV)**			
Hanson et al. (9)	16/32	50.00 (31.89–68.11)	8.44
Raman et al. (11)	417/1,439	28.98 (26.65–31.40)	11.47
Basu et al. (3)	121/286	42.31 (36.51–48.26)	11.09
Gawadia et al. (8)	62/86	72.09 (61.38–81.23)	10.14
Sundararaju et al. (14)	24/141	17.02 (11.22–24.26)	10.65
Zeid et al. (12)	9/25	36.00 (17.97–57.48)	7.87
Kaur et. al. (10)	25/69	36.23 (25.00–48.69)	9.85
Sethi et al. (13)	27/38	71.05 (54.10–84.58)	8.81
Basu et al. (5)	27/145	18.62 (12.64–25.92)	10.67
Basu et al. (6)	66/252	26.19 (20.87–32.08)	11.03
Total (random effects)	794/2,513	38.38 (29.37–47.81)	100
**Study**	**True negative/test negative**	**NPV% (95% CI)**	**Random weight (%)**
**(d) Negative predictive value (NPV)**			
Hanson et al. (9)	85/86	98.84 (93.69–99.97)	9.69
Raman et al. (11)	5,793/6,066	95.5 (94.95–96.01)	11.38
Basu et al. (3)	1,057/1,304	81.06 (78.82–83.15)	11.28
Gawadia et al. (8)	74/76	97.37 (90.82–99.68)	9.5
Sundararaju et al. (14)	139/144	96.53 (92.08–98.86)	10.31
Zeid et al. (12)	27/28	96.43 (81.65–99.91)	7.44
Kaur et. al. (10)	336/344	97.67 (95.47–98.99)	10.92
Sethi et al. (13)	58/64	90.63 (80.7–96.48)	9.22
Basu et al. (5)	67/69	97.1 (89.92–99.65)	9.35
Basu et al. (6)	318/332	95.78 (93.03–97.68)	10.91
Total (random effects)	7,954/8,513	94.83 (90.49–97.91)	100
**Study**	**Sample size**	**AUC (95% CI)**	**Random weight (%)**
**(e) Area under curve (AUC)**			
Hanson et al. (9)	118	0.92 (0.86–0.98)	19.07
Raman et al. (11)	162	0.90 (0.85–0.95)	21.42
Sundararaju et al. (14)	285	0.82 (0.74–0.90)	14.93
Kaur et. al. (10)	413	0.82 (0.73–0.91)	13.53
Basu et al. (5)	214	0.80 (0.74–0.86)	19.07
Basu et al. (6)	584	0.78 (0.68–0.88)	11.98
Total (random effects)	1,776	0.85 (0.80–0.89)	100

*CI, confidence interval*.

### Mortality Among Renal Angina Index >8 vs. Renal Angina Index <8 Patients (Secondary Outcome)

RAI >8 patients (191/1,036) were observed to have a 4.5 times higher odds of mortality as compared with RAI <8 patients (125/2,383) [pooled odds ratio: 4.51 (95% CI: 2.06–9.87) (*I*^2^ = 84.80% (68.70%−92.62%), *p* < 0.0001, random effects; six studies; n = 3,419)] (Table 4; Supplementary Figure 4).

**Table 4:** Meta-analysis of mortality among RAI > 8 vs. RAI < 8 across different studies.

**Study**	**RAI positive**	**RAI negative**	**Odds ratio (95% CI)**	**Random weight (%)**
	**Died/sample size**	**Died/sample size**		
Basu et al. (3)	32/286	49/1,304	3.23 (2.03–5.14)	20.66
Gawadia et al. (8)	21/86	6/76	3.77 (1.43–9.92)	16.62
Sundararaju et al. (14)	59/141	50/144	1.35 (0.84–2.18)	20.57
Kaur et. al. (10)	22/69	7/344	22.54 (9.13–55.63)	17.18
Basu et al. (5)	23/145	0/69	26.67 (1.60–45.85)	5.74
Basu et al. (6)	34/252	13/332	3.83 (1.97–7.42)	19.23
Total (random effects)	191/1,036	125/2,383	4.51 (2.06–9.87)	100

*CI, confidence interval; RAI, renal angina index*.

### Sensitivity Analyses

The *I*^2^ value for sensitivity analyses for all the outcomes (except AUC) was similar to that obtained without excluding the studies based on the previously mentioned parameters (Supplementary Table 4). Also, the proportion (%) of the outcomes obtained from the sensitivity analyses was within the 95% CI of the overall proportion for all the parameters, indicating that the meta-analysis results are robust enough.

### Publication Bias

Funnel plots were made (Supplementary Figure 5) to determine publication bias with the following controls: sensitivity, specificity, PPV, NPV, area under curve, and mortality. Visual inspection of the funnel plot and Egger test for five figures [Supplementary Figure 5B (Egger's test *p*-value = 0.0876); Supplementary Figure 5C (*p* = 0.2294); Supplementary Figure 5D (*p* = 0.9800); Supplementary Figure 5E (*p* = 0.1499); and Supplementary Figure 5F (*p* = 0.1845)] showed a symmetrical distribution indicating no evidence of publication bias. However, Supplementary Figure 5A (Egger's test *p*-value = 0.0432) indicated some evidence of publication bias but was accounted for with the high precision of results seen in the PPV and NPV plots (Supplementary Figures 5C,D, respectively).

## Discussion

RAI has acquired significant attention in the pediatric population in recent years, which has compelled nephrologists to conduct many cohort studies. In this meta-analysis, we examine the mortality and pretest probability of the predictability of RAI in identifying early AKI.

Based on our meta-analysis, the pretest probability for predicting the AKI early in pediatric intensive care unit (PICU) has shown similar data to a study by McGalliard and team. They conducted a prospective cohort study on 657 children analyzing the importance of RAI alone and with NGAL to predict severe AKI (stage 2 or 3) incidence in PICU patients. The study showed a sensitivity of 88%, a specificity of 58%, a PPV of 24%, an NPV of 97%, and an AUC of 0.73. However, when the author combined RAI with a biomarker, NGAL, the AUC was 0.80, suggesting a higher association in predicting severe AKI in PICU patients (17). Similarly, another recent study by Raman *et al*. conducted a single-center retrospective observational study on 7,505 children; their findings showed an AUC for RAI of 0.857. However, when combined with the high risk of AKI (HRA), the AUC was 0.87 (11). In comparison, a retrospective analysis of 390 adult patients admitted to the ICU with septic shock analyzed the ability of SCr alone to identify patients with septic shock at the highest risk for AKI. An increase in SCr alone in the first 12 h of patient stay was not significantly associated with stage 2 or greater AKI (AUC 0.55, 95% CI: 0.47–0.60) (7). Many novel biomarkers, e.g., NGAL, kidney injury molecule 1, cystatin C, and interleukin-18, may help identify early AKI; however, a detailed understanding of the mechanism of kidney injury and the use of more than one biomarker may be required. Some of the challenges faced while using these biomarkers include delay in results (requires at least 5–7 h), lack of specific equipment, and increased cost of treatment (3, 18).

Though several studies have emphasized the significance of RAI, few studies utilized the index in different risk settings to predict AKI within 3 days of PICU admission. Hanson *et al*. enrolled 118 subjects to test the sensitivity of RAI to indicate AKI early in the pediatric AKI in the emergency department. The RAI score demonstrated 94% sensitivity and 99% NPV. Thus, it showed the importance of RAI as an emergency department tool to anticipate AKI and initiate early management (9). Similarly, an interesting study by Huang *et al*. showed the use of RAI in septic shock pediatric patients, where 66 patients with septic shock were studied and analyzed to predict their likelihood of acquiring AKI within 3 days of the PICU admission. The study showed that the RAI score (AUC = 0.78) identified the high-risk AKI better than the earliest elevated baseline SCr levels (AUC = 0.70). However, the combination of RAI with serum lactate (AUC = 0.83) performed superior to both RAI alone and high baseline SCr levels (19). Comparably, Stanski et al. (20) discussed the Pediatric Sepsis Biomarker Risk Model (PERSEVERE) as a stratification tool to estimate the baseline risk of mortality in pediatric septic shock. The authors aimed to assess the efficacy of PERSEVERE in predicting the development of septic shock-associated AKI among children on day 3 of hospital admission. The PERSEVERE biomarkers included C-C chemokine ligand 3, granzyme B, heat shock protein 70 kDa 1B, interleukin-8, and MMP-8. The study concluded that a higher PERSEVERE score was independently associated with increased odds of severe day 3 sepsis-associated AKI (OR, 1.4, 95% CI: 1.2–1.7, *p* < 0.001) (20, 21). Therefore, combining multiple biomarkers and comparing their impact on AKI prediction and the significance of RAI in other high AKI risk situations like septic shock need some exploration. Similarly, Youssef et al. (22) conducted a single-center retrospective study on 53 critically ill children. The study compared the AKI prediction with RAI and cystatin C. There was a higher prediction rate when cystatin C was combined with RAI (accuracy of 96.2 vs. 94.3% (RAI alone) vs. 90.6% (cystatin C alone). These studies have shown that combining various biomarkers with RAI may improve the ability to predict AKI in critically ill children (22).

In accordance with the goals of RAI, a 2019 KDIGO conference entitled “Acute Kidney Injury” suggested incorporating kidney damage biomarkers, biopsy, and imaging into the current KDIGO AKI guidelines. The conference report discussed that with the incorporation of additional factors into AKI diagnoses, physicians will be better equipped to identify, classify, and treat AKI. Such modifications may also potentially allow the diagnosis of AKI to become more unified for children and adults with low muscle mass and low SCr levels. Likewise, while the definition of “preclinical” AKI has been deemed to have evidence of importance, there is a lack of consensus on how to advance diagnostic standards further (21, 23). With further prospective studies of RAI in both adult and pediatric populations, RAI holds the potential to advance preclinical AKI diagnostic measures.

In this meta-analysis, there are some important limitations worth mentioning. When combining data from multiple studies to conduct meta-analysis, it does not overcome the limitations that were inherent in the primary studies design, creating a publication bias. The low PPV in these studies can be recognized as a limitation but explainable due to the low incidence of AKI in the pediatric population. Since the NPV for the studies used in the meta-analysis was significant, the RAI method still proves its effectiveness as an important diagnostic tool.

## Conclusion

The finding of this analysis suggests that RAI is a potential tool in indicating the early AKI events in ICU pediatric patients and also suggesting the high-risk mortality in patients with RAI score of ≥8. Additionally, combining RAI with other biomarkers has shown better AKI predictability. However, more extensive prospective cohort studies are required to determine the predictability of RAI and to diminish heterogeneity in critically ill pediatric populations.

## Data Availability Statement

The original contributions presented in the study are included in the article/Supplementary Material, further inquiries can be directed to the corresponding author.

## Author Contributions

RR, NA, IM, ND, SS, and RC contributed to the conception and design and wrote sections of the manuscript. AT performed the statistical analysis. All authors contributed to manuscript revision and read and approved the submitted version.

## Conflict of Interest

The authors declare that the research was conducted in the absence of any commercial or financial relationships that could be construed as a potential conflict of interest.

## Publisher's Note

All claims expressed in this article are solely those of the authors and do not necessarily represent those of their affiliated organizations, or those of the publisher, the editors and the reviewers. Any product that may be evaluated in this article, or claim that may be made by its manufacturer, is not guaranteed or endorsed by the publisher.

## Supplementary Material

Supplementary Figure 1 PRISMA Flowchart.

Supplementary Figure 2 Forest plot of the meta-analysis of RAI area under the curve across different studies. The lower diamond in the graph represents the pooled estimate.

Supplementary Figure 3 Summary receiver operating characteristic (SROC) graph of 10 included studies based on random effects model. HSROC: Hierarchical summary receiver-operating characteristic. Each data point comes from a different study, not a different threshold. This figure showed that the area under the ROC curve is 0.82 (the point marked with blue), indicating good accuracy of RAI for diagnosis of AKI.

Supplementary Figure 4 Forest plot of the mortality among RAI positive (>8) vs. RAI negative (<8) across different studies. The lower diamond in the graph represents the pooled estimate.

Supplementary Figure 5 Funnel Plots for RAI includes values for all of the included studies providing the respective data: **(A)** sensitivity, **(B)** specificity, **(C)** positive predictive value, **(D)** negative predictive value, **(E)** Area under curve, and **(F)** mortality among RAI positive (>8) vs. RAI negative (<8).
