# Supplementary figures and images for: Re-evaluating Renal Angina Index: An Authentic, Evidence-Based Instrument for Acute Kidney Injury Assessment: Critical Appraisal
Source: Front Pediatr. 2021 Jul 29;9:682672. doi: 10.3389/fped.2021.682672 (PMC8358434; doi:10.3389/fped.2021.682672)

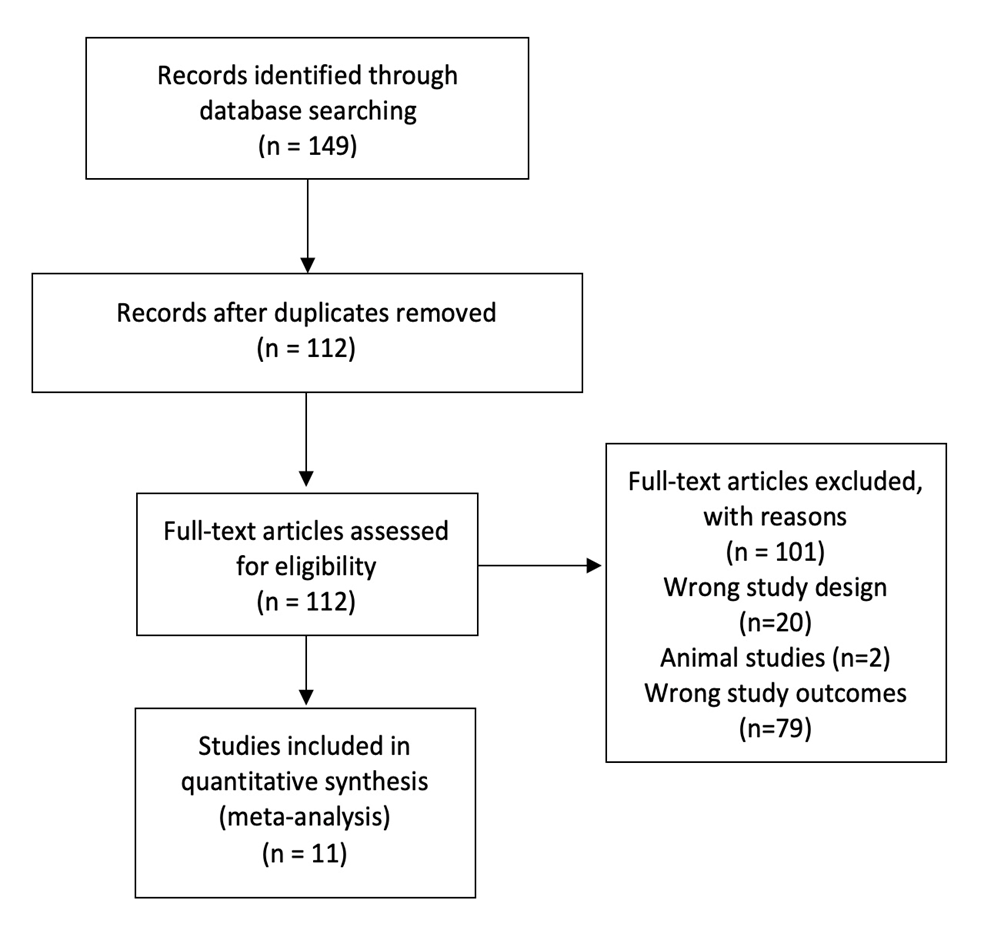

Supplement: Supplementary Figure 1 — PRISMA Flowchart. [file Image_1.TIF]

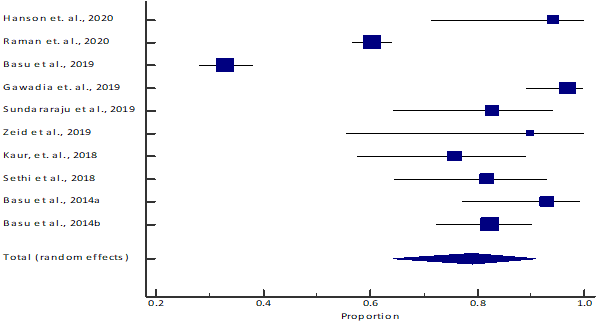

Supplement: Supplementary Figure 2 — Forest plot of the meta-analysis of RAI area under the curve across different studies. The lower diamond in the graph represents the pooled estimate. [file Image_2.TIF]

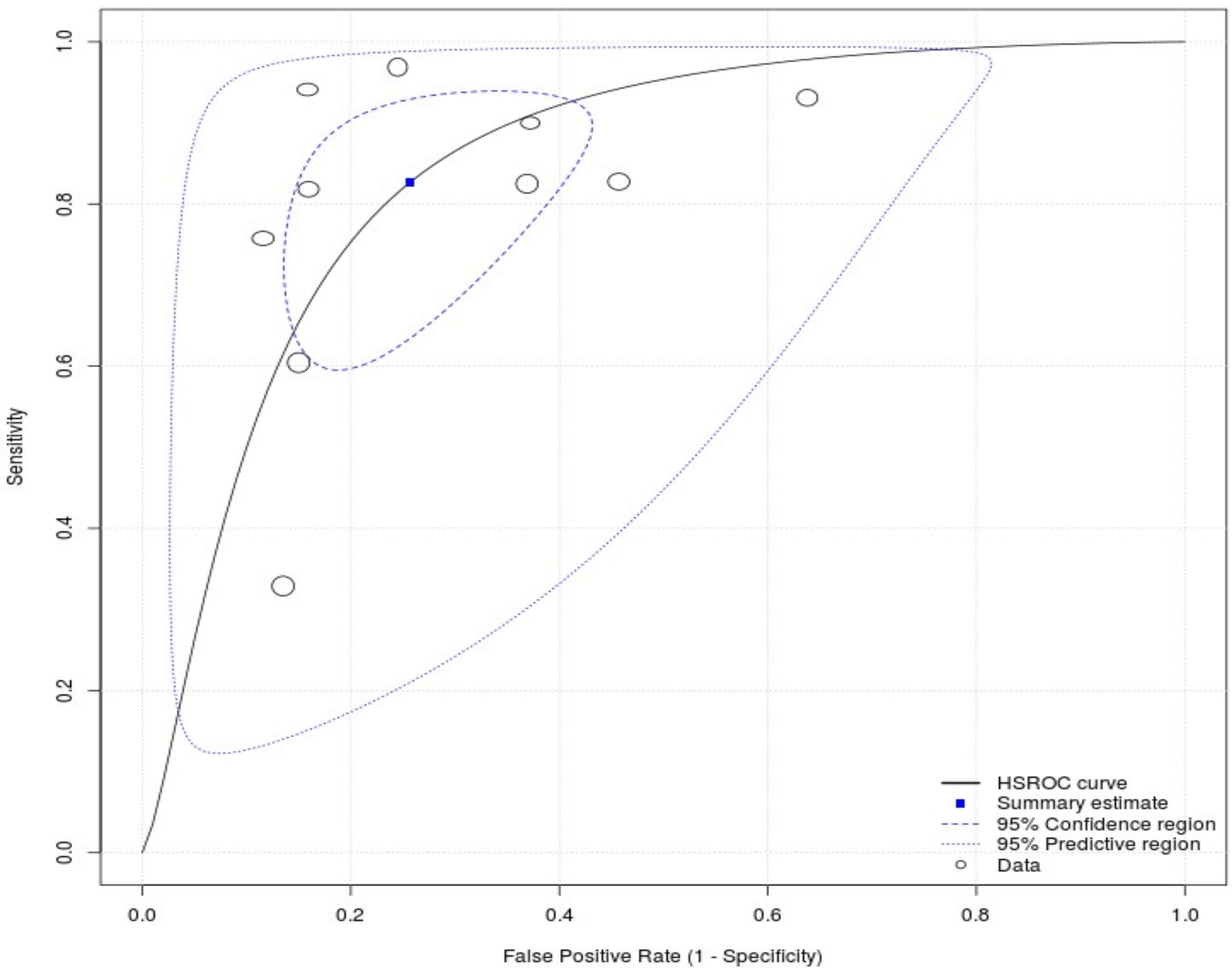

Supplement: Supplementary Figure 3 — Summary receiver operating characteristic (SROC) graph of 10 included studies based on random effects model. HSROC: Hierarchical summary receiver-operating characteristic. Each data point comes from a different study, not a different threshold. This figure showed that the area under the ROC curve is 0.82 (the point marked with blue), indicating good accuracy of RAI for diagnosis of AKI. [file Image_3.JPEG]

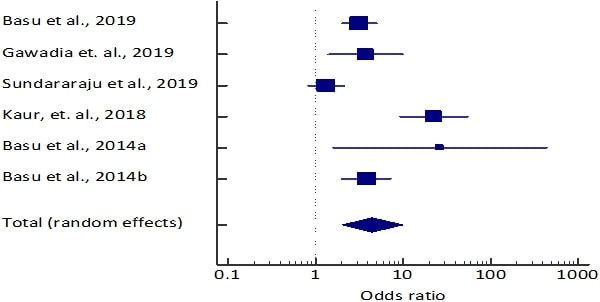

Supplement: Supplementary Figure 4 — Forest plot of the mortality among RAI positive (>8) vs. RAI negative (<8) across different studies. The lower diamond in the graph represents the pooled estimate. [file Image_4.JPEG]

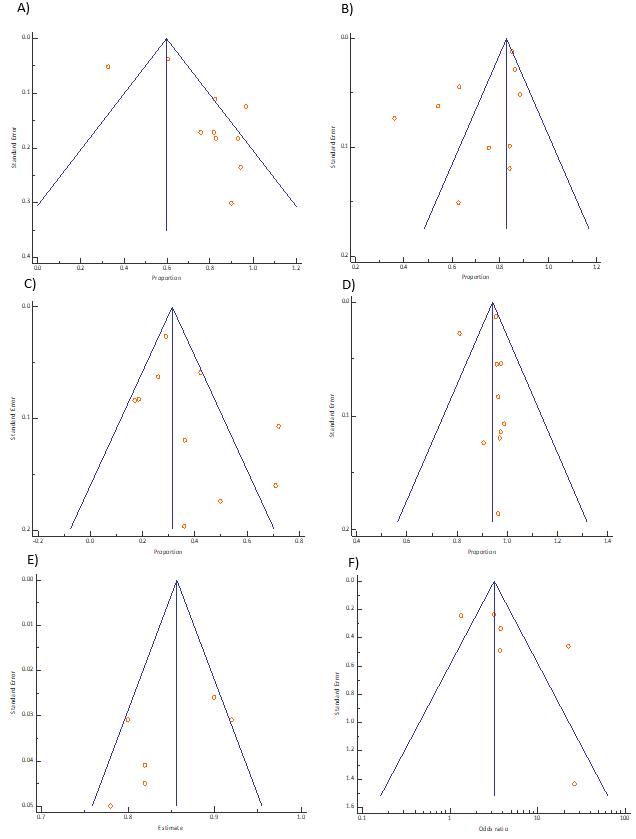

Supplement: Supplementary Figure 5 — Funnel Plots for RAI includes values for all of the included studies providing the respective data: (A) sensitivity, (B) specificity, (C) positive predictive value, (D) negative predictive value, (E) Area under curve, and (F) mortality among RAI positive (>8) vs. RAI negative (<8). [file Image_5.PNG]
