# Supplementary material for: Re-evaluating Renal Angina Index: An Authentic, Evidence-Based Instrument for Acute Kidney Injury Assessment: Critical Appraisal
Source: Front Pediatr. 2021 Jul 29;9:682672. doi: 10.3389/fped.2021.682672 (PMC8358434; doi:10.3389/fped.2021.682672)
Supplement: Supplementary file 6 [file Data_Sheet_1.docx]

**Supplementary data**

**Table S1:** A systematic review PICO table

| **Criteria** | **Inclusion** | **Exclusion** |
| --- | --- | --- |
| **Population** | Pediatric patients with high-risk AKI | Adult population |
| **Intervention** | Patients with RAI measurement | Did not measure RAI |
| **Comparison** | No Controls | - |
| **Outcome** | To see the prognostic aspect of early prediction of AKI in pediatric population via renal angina index versus serum creatinine. | - |
| **Study types** | Prospective and Retrospective studies | Case Reports, Systematic reviews, Literature reviews |

AKI, acute kidney injury; RAI, renal angina index.

| **Table S2:** Prisma checklist | | | |
| --- | --- | --- | --- |
| **Section and topic** | **#** | **Checklist item** | **Inclusion**  **(page #)** |
| **TITLE** | | |  |
| Title | 1 | Identify the report as a systematic review and meta-analysis of individual participant data. | 1 |
| **Abstract** | | |  |
| Structured summary | 2 | Provide a structured summary including, as applicable: background; objectives; data sources; study eligibility criteria, participants, and interventions; study appraisal and synthesis methods; results; limitations; conclusions and implications of key findings; systematic review registration number.: | 2 |
| **INTRODUCTION** | | |  |
| Rationale | 3 | Describe the rationale for the review in the context of what is already known. | 3 |
| Objectives | 4 | Provide an explicit statement of questions being addressed with reference to participants, interventions, comparisons, outcomes, and study design (PICOS). | 4 |
| **METHODS** | | |  |
| Protocol and registration | 5 | Indicate if a review protocol exists, if and where it can be accessed (e.g., Web address), and, if available, provide registration information including registration number. | N/A |
| Eligibility criteria | 6 | Specify study characteristics (e.g., PICOS, length of follow-up) and report characteristics (e.g., years considered, language, publication status) used as criteria for eligibility, giving rationale. | 5 |
| Information sources | 7 | Describe all information sources (e.g., databases with dates of coverage, contact with study authors to identify additional studies) in the search and date last searched. | 5 |
| Search | 8 | Present full electronic search strategy for at least one database, including any limits used, such that it could be repeated. | 5 |
| Study selection | 9 | State the process for selecting studies (i.e., screening, eligibility, included in systematic review, and, if applicable, included in the meta-analysis). | 5 |
| Data collection process | 10 | Describe method of data extraction from reports (e.g., piloted forms, independently, in duplicate) and any processes for obtaining and confirming data from investigators. | 5,6 |
| Data items | 11 | List and define all variables for which data were sought (e.g., PICOS, funding sources) and any assumptions and simplifications made. | 5,6 |
| Risk of bias in individual studies | 12 | Describe methods used for assessing risk of bias of individual studies (including specification of whether this was done at the study or outcome level), and how this information is to be used in any data synthesis. | 5,6 |
| Summary measures | 13 | State the principle summary measures (e.g., risk ratio, difference in means). | 5,6 |
| Synthesis of results | 14 | Describe the methods of handling data and combining results of studies, if done, including measures of consistency (e.g., I2) for each meta-analysis. | 5,6 |
| Risk of bias across studies | 15 | Specify any assessment of risk of bias that may affect the cumulative evidence (e.g., publication bias, selective reporting within studies). | 5,6 |
| Additional analyses | 16 | Describe methods of additional analyses (e.g., sensitivity or subgroup analyses, meta-regression), if done, indicating which were pre-specified. | 6 |
| **RESULTS** | | | |
| Study selection | 17 | Give numbers of studies screened, assessed for eligibility, and included in the review, with reasons for exclusions at each stage, ideally with a flow diagram. | 7 |
| Study characteristics | 18 | For each study, present characteristics for which data were extracted (e.g., study size, PICOS, follow-up period) and provide the citations. | 7 |
| Risk of bias within studies | 19 | Present data on risk of bias of each study and, if available, any outcome-level assessment (see Item 12). | N/A |
| Results of individual studies | 20 | For all outcomes considered (benefits or harms), present, for each study: (a) simple summary data for each intervention group and (b) effect estimates and confidence intervals, ideally with a forest plot. | 7,8 |
| Synthesis of results | 21 | Present results of each meta-analysis done, including confidence intervals and measures of consistency | 7,8 |
| Risk of bias across studies | 22 | Present results of any assessment of risk of bias across studies (see Item 15). | 7,8 |
| Additional analysis | 23 | Give results of additional analyses, if done (e.g., sensitivity or subgroup analyses, meta-regression [see Item 16]). | 8 |
| **DISCUSSION** | | | |
| Summary of evidence | 24 | Summarize the main findings including the strength of evidence for each main outcome; consider their relevance to key groups (e.g., health care providers, users, and policy makers). | 9-11 |
| Limitations | 25 | Discuss limitations at study and outcome level (e.g., risk of bias), and at review level (e.g., incomplete retrieval of identified research, reporting bias) | 11 |
| Conclusions | 26 | Provide a general interpretation of the results in the context of other evidence, and implications for future research | 12 |
| **FUNDING** | | | |
| Funding | 27 | Describe sources of funding for the systematic review and other support (e.g., supply of data); role of funders for the systematic review. | 12 |

**Table S3:** Quality Assessment for all the included studies

| **Parameters** | **Studies** | | | | | | | | | | |
| --- | --- | --- | --- | --- | --- | --- | --- | --- | --- | --- | --- |
|  | **Gawadia et. al 2019** | **Hanson et. al 2020** | **Kaur, et. al 2018** | **Raman et. al 2020** | **Zeid et al. 2019** | **Basu et al. 2019** | **Basu et al. 2014** | **Sethi et al. 2018** | **Sundararaju et al. 2019** | **Roy et al. 2019** | **Basu et al. 2014** |
| **Research Question** | Y | Y | Y | Y | Y | Y | Y | Y | Y | Y | Y |
| **Study population clearly specified and defined?** | Y | Y | Y | Y | Y | Y | Y | Y | Y | Y | Y |
| **The participation rate of eligible persons at least 50%?** | Y | N | Y | N | Y | Y | Y | N | Y | Y | Y |
| **Groups recruited from the same population and uniform eligibility criteria** | Y | Y | Y | Y | Y | Y | Y | Y | Y | Y | Y |
| **Sample size justification** | Y | Y | Y | Y | Y | Y | Y | Y | Y | Y | Y |
| **Exposure assessed prior to outcome measurement** | Y | Y | Y | Y | Y | Y | Y | Y | Y | Y | Y |
| **Sufficient timeframe to see an effect** | Y | Y | Y | Y | Y | Y | Y | Y | Y | Y | Y |
| **Different levels of exposure of interest** | Y | Y | Y | Y | Y | Y | Y | Y | Y | Y | Y |
| **Exposure measures and assessment** | Y | Y | Y | N | Y | Y | Y | N | Y | N | Y |
| **Repeated exposure assessment** | N/A | N/A | N/A | N/A | N/A | N/A | N/A | N/A | N/A | N/A | N/A |
| **Outcome measures** | Y | Y | Y | Y | Y | Y | Y | Y | Y | Y | Y |
| **Blinding of outcome assessors** | N/A | N/A | N/A | N/A | N/A | N/A | N/A | N/A | N/A | N/A | N/A |
| **Follow-up rate** | Y | Y | Y | Y | Y | Y | Y | Y | Y | Y | Y |
| **Statistical analysis** | Y | Y | Y | Y | Y | Y | Y | Y | Y | Y | Y |
| **Overall Outcome (Good, Fair, Poor)** | 12 | 11 | 12 | 10 | 12 | 12 | 12 | 10 | 12 | 11 | 12 |

Y: Yes; N: No

**Table S4:** Sensitivity analyses for different outcomes

| **Sensitivity analyses based on type of study** | | | | | | | | | | | |
| --- | --- | --- | --- | --- | --- | --- | --- | --- | --- | --- | --- |
| Outcome | Type of study | | Event / Sample size | | Pooled value (95% CI) | | I^2^ (95% CI) | | N | | p value |
| Sensitivity (95% CI) | Prospective | | 284/554 | | 79.76% (51.75% - 97.29%) | | 96.95% (95.38% - 97.98%) | | 7 | | <0.0001 |
| Specificity (95% CI) | Prospective | | 1,776/2,169 | | 77.53% (66.89% - 86.61%) | | 95.62% (93.04% - 97.24%) | | 7 | | <0.0001 |
| PPV (95% CI) | Prospective | | 284/677 | | 45.88% (30.47% - 61.71%) | | 93.44% (88.94% - 96.11%) | | 7 | | <0.0001 |
| NPV (95% CI) | Prospective | | 1,776/2,046 | | 94.39% (86.64% - 98.92%) | | 95.87% (93.50% - 97.38%) | | 7 | | <0.0001 |
| AUC (95% CI) | Prospective | | 978 | | 0.88 (0.84 - 0.92) | | 51.35% (0.00% - 83.92%) | | 4 | | 0.1038 |
| Mortality [Odds ratio (95% CI)]+ | Prospective | | 134/582 vs. 112/1,868 | | 4.2 (1.50 - 11.53) | | 90% (77.33% - 95.59%) | | 4 | | <0.0001 |
| **Sensitivity analyses based on Sample size** | | | | | | | | | | | |
| Outcome | Sample size | | Event / Sample size | | Pooled value (95% CI) | | I^2^ (95% CI) | | N | | p value |
| Sensitivity (95% CI) | >33 | | 666/1,202 | | 70.53% (45.50% - 90.27%) | | 98.39% (97.41% - 99.00%) | | 4 | | <0.0001 |
| Specificity (95% CI) | >221 | | 7,643/9,177 | | 76.88% (66.95% - 85.49%) | | 98.38% (97.55% - 98.93%) | | 5 | | <0.0001 |
| PPV (95% CI) | >114 | | 655/2,263 | | 26.69% (19.79% - 34.20%) | | 90.67% (81.16% - 95.38%) | | 5 | | <0.0001 |
| NPV (95% CI) | >115 | | 7,643/8,190 | | 94.02% (87.08% - 98.41%) | | 98.44% (97.66% - 98.96%) | | 5 | | <0.0001 |
| AUC (95% CI) | >250^ | | 1,282 | | 0.81 (0.76 - 0.86) | | 0% (0.00% - 85.67%) | | 3 | | 0.7913 |
| Mortality [Odds ratio (95% CI)]+ | >350 | | 88/607 vs. 69/1,980 | | 6.13 (2.21 - 17.00) | | 86.11% (59.74% - 95.21%) | | 3 | | 0.0007 |
| **Sensitivity analyses based on quality of study** | | | | | | | | | | | |
| Outcome | Study quality | Event / Sample size | | Pooled value (95% CI) | | I2 (95% CI) | | n | | p value | |
| Sensitivity (95% CI) | Good | 334/613 | | 79.97% (53.29% - 96.95%) | | 97.32% (96.01% - 98.20%) | | 7 | | < 0.0001 | |
| Specificity (95% CI) | Good | 2,018/2,688 | | 67.92% (52.37% - 81.66%) | | 98.31% (97.62% - 98.80%) | | 7 | | < 0.0001 | |
| PPV (95% CI) | Good | 334/1,004 | | 34.78% (22.51% - 48.17%) | | 94.39% (90.75% - 96.59%) | | 7 | | < 0.0001 | |
| NPV (95% CI) | Good | 2,018/2,297 | | 94.71% (87.70% - 98.88%) | | 96.25% (94.16% - 97.59%) | | 7 | | < 0.0001 | |
| AUC (95% CI) | Good | 1,658 | | 0.83 (0.78 - 0.88) | | 54.88% (0.00% - 83.35%) | | 5 | | < 0.0646 | |
| Mortality [Odds ratio (95% CI)]+ | Good | 191/979 vs. 125/2,269 | | 4.51 (2.06 - 9.87) | | 84.80% (68.70% - 92.62%) | | 6 | | < 0.0001 | |

^Fixed effect model, all else random effect model

+ Odds ratio for AKI mortality among RAI positive vs. RAI negative

N= number of studies

Sample size based on median values for sensitivity, specificity, PPV, and NPV across all the studies; and median values for the studies for AUC and mortality. AUC, area under the curve; NPV, negative predictive value; PPV, positive predictive value.

**Figure S1:** PRISMA Flowchart.

**Figure S2:** Forest plot of the meta-analysis of RAI area under the curve across different studies. The lower diamond in the graph represents the pooled estimate.

**Figure S3:** Summary receiver operating characteristic (SROC) graph of 10 included studies based on random effects model. HSROC: Hierarchical summary receiver-operating characteristic

Each data point comes from a different study, not a different threshold

This figure showed that the area under the ROC curve is 0.82 (the point marked with blue), indicating good accuracy of RAI for diagnosis of AKI.

**Figure S4:** Forest plot of the mortality among RAI positive (>8) *vs.* RAI negative (<8) across different studies. The lower diamond in the graph represents the pooled estimate.

**Figure S5**: Funnel Plots for RAI includes values for all of the included studies providing the respective data: A) sensitivity, B) specificity, C)positive predictive value, D) negative predictive value, E) Area under curve, and F) mortality among RAI positive (>8) *vs.* RAI negative (<8).
